# Ocular Manifestations of Chikungunya Infection: A Systematic Review

**DOI:** 10.3390/pathogens11040412

**Published:** 2022-03-29

**Authors:** Liziane Cristine Malaquias da Silva, Fernanda da Silva Platner, Lauany da Silva Fonseca, Virgílio Frota Rossato, Dian Carlos Pereira de Andrade, João de Sousa Valente, Susan Diana Brain, Elizabeth Soares Fernandes

**Affiliations:** 1 Programa de Pós-Graduação em Biotecnologia Aplicada à Saúde da Criança e do Adolescente, Faculdades Pequeno Príncipe, Av. Iguaçu No 333, Curitiba 80230-020, PR, Brazil; lizianecms@gmail.com (L.C.M.d.S.); fsilvaplatner@gmail.com (F.d.S.P.); lauanyfonseca@outlook.com (L.d.S.F.); virgiliofrota@gmail.com (V.F.R.); 2 Instituto de Pesquisa Pelé Pequeno Príncipe, Av. Silva Jardim No 1632, Curitiba 80240-020, PR, Brazil; diancarlospa@gmail.com; 3 Vascular Biology and Inflammation Section, School of Medicine, King’s College London, London SE1 9NH, UK; joao.de_sousa_valente@kcl.ac.uk (J.d.S.V.); sue.brain@kcl.ac.uk (S.D.B.)

**Keywords:** Chikungunya infection, ocular manifestations, ocular symptoms, systematic review

## Abstract

The Chikungunya virus (CHIKV) can cause long lasting symptoms and manifestations. However, there is little information on which ocular ones are most frequent following infection. We performed a systematic review (registered in the International Prospective Register of Systematic Reviews; no CRD42020171928) to establish the most frequent ocular manifestations of CHIKV infection and their associations with gender and age. Articles published until September 2020 were selected from PubMed, Scielo, Cochrane and Scopus databases. Only studies with CHIKV-infected patients and eye alterations were included. Reviews, descriptive studies, or those not investigating the human ocular manifestations of CHIKV, those with patients with other diseases and infections, abstracts and studies without relevant data were excluded. Twenty-five studies were selected for inclusion. Their risk of bias was evaluated by a modified Newcastle-Ottawa scale. The most frequent ocular symptoms of CHIKV infection included ocular pain, inflammation and reduced visual acuity, whilst conjunctivitis and optic neuritis were the most common manifestations of the disease. These occurred mostly in individuals of 42 ± 9.5 years of age and woman. The few available reports on CHIKV-induced eye manifestations highlight the need for further research in the field to gather more substantial evidence linking CHIKV infection, the eye and age/gender. Nonetheless, the data emphasizes that ocular alterations are meaningful occurrences of CHIKV infection which can substantially affect quality of life.

## 1. Introduction

The Chikungunya virus (CHIKV) is an arbovirus transmitted through the bite of the female mosquito of the Aedes spp. family [1]. In the 1950’s, the first outbreak was observed in Tanzania, in patients who reported a hypothermia accompanied by intense joint pain [2]. Over the next fifty years, outbreaks of the disease were registered in Thailand (1950s’ and 1960s’) and India (1960s’ and 1970s’). Since 2004, the CHIKV infection has spread in many countries of Africa, Asia, Europe and especially in the Americas [3]. According to the Pan American Health Organization (PAHO), 1.7 million cases of the disease have been reported up to September 2015. Of these, >770,000 cases were confirmed in Brazil from 2013 to 2017 [4]. The European Centre of Disease Prevention and Control (ECDC) indicated that in first 6-months of 2021, the global reach of the disease was of 85,304 notified cases [5].

Diagnosis is sometimes difficult as the CHIKV infection presents symptoms, which are similar to those caused by other arboviruses (dengue, Zika and Mayaro) [6]. Most of the infected individuals are symptomatic. The acute phase of the disease (up to three-weeks post-infection) is characterized by a range of nonspecific symptoms such as high fever (>39 °C), headache, fatigue, rash, myalgias and arthralgias. Of those, the most prevalent is a severe and often debilitating pain and swelling of the joints [7]. The post-acute phase (which lasts from the third week for up to three months following infection) is characterized by resolution of the acute phase symptoms, except for persistent polyarthritis, often observed as joint stiffness, pain and oedema [8]. The available data varies with the population studied and the methodologies used. Usually, >50% of the patients infected with CHIKV in the Americas develop the chronic phase of the disease [9], suffering especially from polyarthralgia. The chronic phase can last from months to years, markedly decreasing the quality of life of the patients [10].

Besides causing joint pain, the virus can also affect other organs and systems including the nervous and cardiovascular systems, skin and kidneys [11]. Prevalence studies have been useful to aid the public health systems to define surveillance policies and design the better management of infectious diseases [12]. Reports indicate that the eyes can be affected by CHIKV; however, the prevalence and the most common types of ocular manifestations triggered by the disease are unclear, as well as their associations with population characteristics. Indeed, to date, there are few reports investigating the prevalence of ocular manifestations in CHIK-infected patients. Therefore, we aimed to conduct a systematic review of the literature to identify the most prevalent ocular manifestations of CHIKV infection and their possible associations with population characteristics such as gender and age.

## 2. Results

### 2.1. Selection of Studies

**Figure 1:**
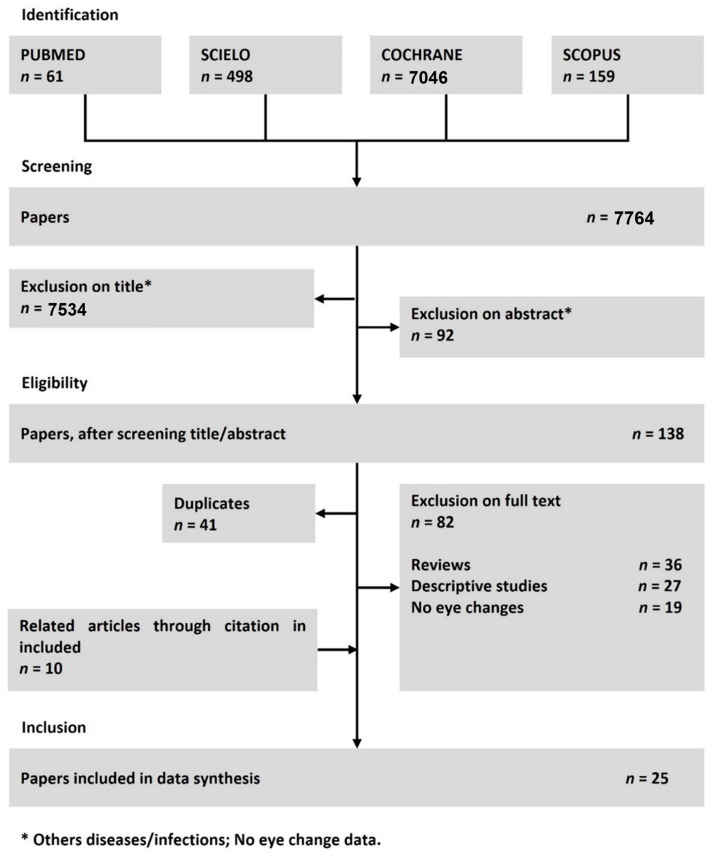
Flow diagram of the search process and study selection.

The flow of study selection is depicted in Figure 1. The primary database search resulted in 7764 manuscripts of which, 7534 were excluded by title and 92 by abstract content. The remaining 138 papers were analysed for eligibility. Next, 123 articles were excluded—41 due to duplication and 82 for not satisfying the established selection criteria after full text review. The remaining 15 studies were selected for inclusion in the review. Additionally, following bibliography search in the selected articles, 10 other studies were manually included after meeting the eligibility criteria. These 25 reports were included in the data extraction phase.

### 2.2. Geographical, Age and Gender Distribution

Of the articles included in this study, 15 contained data from India (60%), two were reports from France (8%) and two from Puerto Rico (8%). The remaining 06 studies (20%) were from Chile, Italy, Mexico, Brazil, Bangladesh and Germany (one from each country). The 25 analysed reports included a total of 6831 patients diagnosed with CHIKV infection. Of those, 1824 (26.7%) presented with at least one type of ocular symptom or manifestation (Table 1). Twelve of the selected studies contained more than 100 patients each, with two of them containing more than 1000 patients. Amongst the 25 selected articles, 18 did not contain data on the mean age of the patients—only presenting the age groups included in the study and did not discriminate gender. Based on the remaining seven studies, the mean age of the patients was calculated and found to be equal to 42 ± 9.5 years, with adults being the most affected. Furthermore, a total of 77 patients had their gender described in these seven studies; of those, 33 were men (43%) and 44 women (57%).

### 2.3. Ocular Alterations

Analysis of all the 25 studies identified three main ocular symptoms in CHIKV patients. Ocular inflammation was the most frequently reported symptom; it was noted in 1331 out of 3998 patients (33%) infected by the virus distributed in 11 out of 25 studies (Table 2). Visual defects were the second most observed symptoms, found in 10 out of 25 selected studies, with 23% (137 out of 575) of the infected patients presenting with this symptom (Table 3). Eye pain was also observed in a relevant number of the selected articles (6 out 25 studies) and affected 19% (306 out of 1545) of the CHIKV patients presented in those studies (Table 4).

Of all the reports describing visual defects, half provided detailed information on patient’s gender and age, with data on 34 out of 306 patients being available. The mean age of CHIKV patients who presented visual defects was of 40 ± 5 years, and the gender distribution was as follows: 12 men (35%) and 22 women (65%).

Of all the reports describing visual defects, half provided detailed information on patient’s gender and age, with data on 34 out of 306 patients being available. The mean age of CHIKV patients who presented visual defects was of 40 ± 5 years, and the gender distribution was as follows: 12 men (35%) and 22 women (65%). Of the studies reporting ocular inflammation, only three displayed specific information on gender and age. In these studies, of the 1331 patients presenting eye inflammation, only 40 had these data available: 19 were men (1.4%) and 21 were women (1.6%), and the mean age of the population was 44 ± 10 years. In regards of eye pain, only three reports contained information on gender and mean age, these related to 13 out of the 137 patients: five men (4%) and eight women (6%), with mean age of 10 ± 10 years.

In addition to symptoms, the articles also presented information on the type of ocular manifestations as a result of CHIKV infection. These included corneal involvement, conjunctivitis, episcleritis, optic neuritis and uveitis.

Sixteen of the selected articles described at least one type of ocular manifestation (Table 5). In a total of 2267 CHIKV-infected patients, 348 exhibited ocular manifestations. Conjunctivitis was the most frequently observed, affecting 201 individuals. Eighty-seven subjects were diagnosed with optic neuritis following infection, whilst fifty-six presented uveitis. Corneal involvement was observed in three patients, and episcleritis was noted in only one patient.

### 2.4. Analysis of Risk of Bias

**Figure 2:**
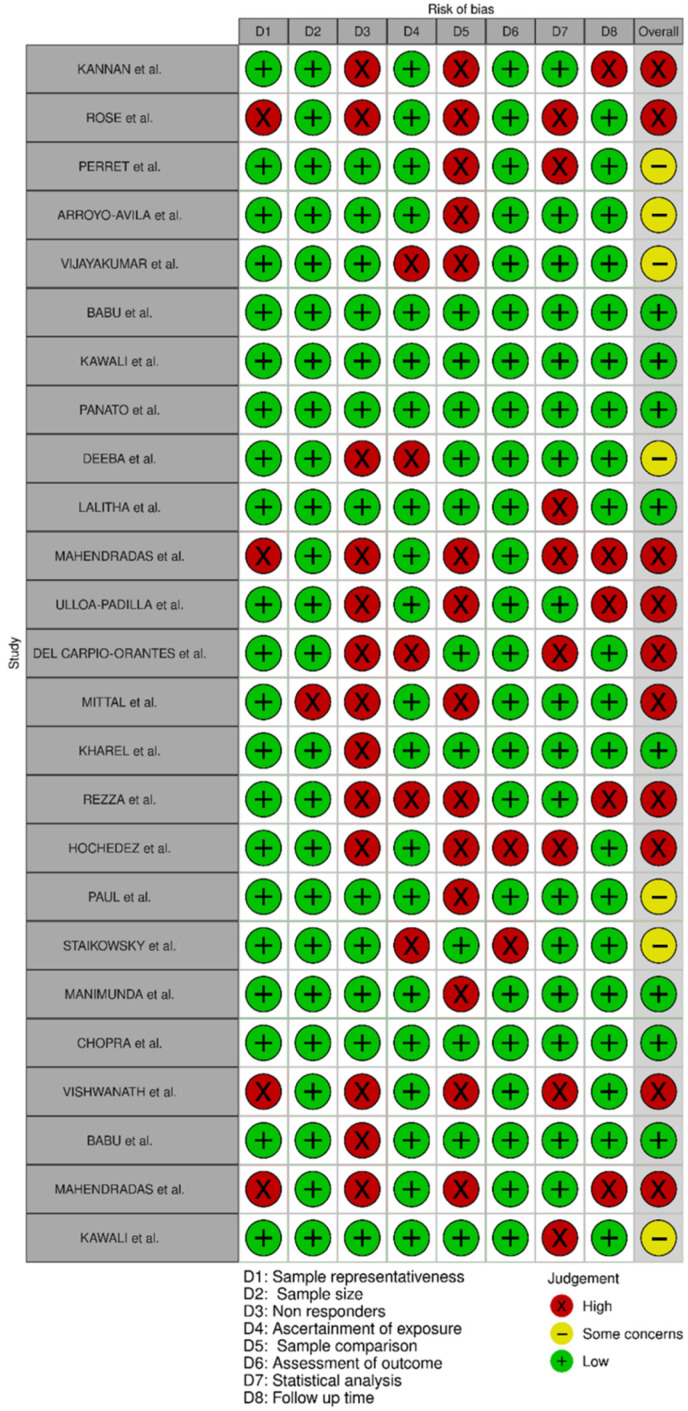
Quality assessment of the selected research papers using a modified version of the Newcastle-Ottawa scale for analysis of risk of bias (RoB). Green/positive indicates items that were judged as low RoB; Yellow/negative indicates items that were judged with some concerns (moderate RoB). RoB; Red/X indicates items that were judged as high RoB [13,14,15,16,17,18,19,20,21,22,23,24,25,26,27,28,29,30,31,32,33,34,35,36,37].

ROB analysis of the 25 selected manuscripts demonstrated that eight of manuscripts are of low risk, another eight are of moderate and nine of high risk of bias (Figure 2). In the first domain—denoted as sample representativeness—four articles were classified as of high risk either for not being representative of the target population or for lacking information on this parameter. In the second domain—sample size—only one study was at high risk of bias, where the sample size was not justified. Thirteen articles were classified as of high risk of bias in the third domain—non responders, as this information was not presented. Four articles were considered as of high risk in the fourth domain of the bias analysis—ascertainment of exposure—for not describing the validation tool used confirm exposure to the virus. In the fifth domain—sample comparison—most of the studies were classified as high risk as 14 manuscripts did not have a control group for comparison of outcomes. The sixth domain—assessment of outcome—indicated three studies were of high risk of bias due to either self-report or lack of information. Additionally, eight articles were classified as high risk of bias in the seventh domain—statistical analysis—due to either incomplete or absent analysis. Finally, in the last domain of the bias analysis—follow up time—five studies were of high risk, including one where the follow up time was insufficient for the resolution of the symptoms and manifestations to occur.

## 3. Discussion

Herein, we attempted to identify the most common ocular symptoms and manifestations of CHIKV infection, as well as their possible associations with individual characteristics such as gender and age. The primary search retrieved 7764 articles but only 25 matched all the criteria necessary to be included in this review. RNA viruses that cause infectious diseases can cause a wide spectrum of ocular disorders [38]. Although the most common symptoms of CHIKV infection are fever, headache, rash and polyarthralgia, the virus appears to present tropism for the nervous system in the ocular tissue [39]. In fact, eye alterations are recognized as important complications of Chikungunya fever, although their exact characteristics had not previously been defined [40,41].

In total, the 25 selected articles included 6831 patients diagnosed with CHIKV infection. Of those, 1814 presented with at least one type of ocular manifestation or symptom, meaning that the virus affected the ocular system in 26% of the infected individuals included in this review. The data indicates that CHIKV infection may cause eye alterations in a quarter of the infected population. This frequency implies the relevance of these manifestations, and the possible burden they can imply in the health system and in the patient’s quality of life [42].

Our study identified that ocular pain and inflammation, as well as visual defects were the ocular symptoms most associated with CHIKV infection. According to the literature, retro-orbital pain is a frequent symptom of the viral infection which can be present in the acute phase of the disease and sometimes become persistent following disease resolution [42]. Furthermore, the exact temporal relationship between the appearance of typical symptoms such as fever and headache and of ocular pain is unclear. Its appearance can vary depending on the ocular structure affected and the degree of ocular involvement [43]. A range of ocular structures can become inflamed during CHIKV infection, including the choroid, uvea, nerve, vitreous, retina and retinal vessels [44]. Even though each eye structure has its own physiology, redness of the eye has been considered as the main indicative of virus-induced eye inflammation [44,45,46].

Different types of visual defects have been linked to CHIKV infection. We identify decreased visual acuity as the major visual defect linked to the virus. Moderate to severe reduction in visual acuity has been observed in CHIKV-infected patients [47,48]. Reduced visual acuity has also been reported for other arbovirus infections (Zika and dengue) [49,50]. Blurred vision was described as one of the visual defects found in infected patients in three of selected articles in our study; this symptom was also suggested as one of the most frequent visual defects associated with the disease [51,52].

We were also able to identify the most common ocular manifestations of CHIKV infection; these included corneal involvement, conjunctivitis, episcleritis, optic neuritis and uveitis. Corneal involvement was found in three patients. The corneal tissue (mainly corneal fibroblasts and corneal endothelium) is suggested to be the eye structure with the greatest viral tropism [39]. Indeed, viral RNA was detected in the eye tissue of patients and corneal grafts from potential donors—even in one with negative serology [53]. Keratitis is the most common form of corneal involvement [40].

Conjunctivitis was the most prevalent ocular manifestation found in the articles included in this study, affecting 201 patients. It is observed in the acute phase of the disease [54] and it is the most common eye manifestation in travellers who go to countries where this arbovirus is emergent [55]. Due to its self-limiting and non-specific nature it is possible that this manifestation is currently under-reported [40].

Episcleritis was described in one patient of the analysed studies; inflammation in the episcleral, although rare, has been previously linked to CHIKV infection [47]. This manifestation has a good prognosis and apparently begins 4 to 6 weeks after the onset of CHIKV fever [56].

Optic neuritis was the second most frequently reported ocular manifestation in the articles included in this review. According to the literature, nerve inflammation is a common ocular complication of the infection, being one of the main causes of vision loss in the patients [57]. It can occur at the beginning of the infection or after, following disease progression [58]. Although its outcome is usually favourable following corticoid treatment, cases of optic neuritis in CHIKV patients can result in blindness [59].

Uveitis was another ocular manifestation identified in our study, with six patients presenting this condition. Patients with uveitis may present vision loss, scotoma, colour vision and peripheral field defects [60]. This manifestation can occur either in the acute or chronic phase of the disease [56]. Anterior uveitis (in the form of retinitis, choroiditis or neuroretinitis) is more common than posterior uveitis [61] which can be acute in immunocompromised patients [62].

Of note, most of the studies included for analysis contained data from patients who presented ocular alterations in the acute and post-acute phases of the disease, with only 2 out of the 25 studies [20,22] presenting data from chronic eye alterations such as reduced visual acuity, following CHIKV infection. Therefore, we were unable to establish how long each ocular alteration may last for following infection. However, it is important to highlight that loss of vision and acuity in chronic disease, and even blindness [22,59,60] were reported in individuals who were infected by the virus suggesting it can cause long lasting and sometimes irreversible damage to the eye structure.

All the 25 articles included in our study were submitted to an analysis of risk of bias. Eight articles were classified as of low risk of bias; of those, four met all the criteria in the established domains, and the other four failed in one of the domains. Moderate risk of bias was attributed to eight studies; five of them did not meet the criteria in two of the domains, while four of them failed in one domain which was more critical for the quality of the publications. Nine articles were at high risk of bias; three of then failed in three domains of analysis and six of them did not meet the criteria in four or more domains.

This RoB assessment shows that most of the articles included in the present study lacked quality in regards of the available data. The domain of sample comparison was the greatest concern as 14 out of the 25 selected studies did not present data from control groups in comparison with CHIKV infected patients.

Other aspect which deserves attention is that the ROB analysis identified that 32% of the articles (8 out of 25) presented incomplete or no details of statistical analysis. Appropriate statistical analysis is detrimental to the quality of data, interpretation of the results and conclusions, as well as to the subsequent reproducibility of the study [63]. The confirmation of exposure to the virus also deserves attention as five articles [17,21,25,28,31] used the clinical symptoms only instead of laboratorial tests as the method of diagnosis for CHIKV infection. Ocular symptoms and ocular manifestations can be linked to more than one type of arbovirus and considering that many of them are transmitted by the same vector—the *Aedes* ssp. mosquito in the case of Dengue, CHIK and Zika viruses—and have very similar presentations [41], an accurate diagnosis is essential to avoid mistakes such as under- or super-notification of cases, wrong treatment and inappropriate connections between such diseases and their manifestations.

We therefore compared the data obtained from the five studies (1434 patients) lacking laboratorial confirmation of CHIKV infection with those from all the 25 studies included in this review. Ocular inflammation and conjunctivitis were respectively the most common symptom and manifestation of the disease found in the five studies in which diagnosis was based on clinical diagnosis only. Both conjunctivitis and ocular inflammation were also amongst the most common manifestations and symptoms of the disease, respectively, when analysing all 25 studies together. Therefore, the lack of laboratorial diagnosis did not affect the identification of eye alterations. Since the studies with no laboratorial confirmation of CHIKV did not contain information separated by either gender or age of the patients, they did not affect the analysis of these data.

The distribution of CHIKV infection cases around the world is directly related to the mobility of infected individuals to areas in which the mosquito vectors are present and able to easily reach to human [64]. Thus, findings were extracted from studies performed mainly from countries with tropical climate (21 out of 25 of the selected articles), with the other four studies containing data from European countries with a temperate climate.

A Brazilian study suggested than 65% of the infections occur in individuals aged between 20 and 59 years, mostly women [65]. Thus, an objective of this systematic review was to determine the associations between characteristics such as gender and age with the prevalence and types of ocular symptoms or manifestations. Half of the articles containing information on ocular pain did not present this detailed information. Only three of the 11 articles indicating ocular inflammation, 5 out of 10 studies which focused on visual defects, and 5 out of the 16 publications discussing ocular manifestations of the disease presented sufficient data of individual characteristics. With the available information from a total of 77 patients, it was possible to observe that eye alterations due to CHIKV occur mostly in patients of 42 ± 9.5 years of age, and women (57%). It is possible thus, that women are not only more prone to infection, but also to the deleterious effects of the virus to the ocular system. However, the lack of substantial and detailed evidence in the literature made it impossible to establish a trustful link between the characteristics of the affected populations and the ocular alterations caused by the virus.

## 4. Methods

### 4.1. Search Strategy

**Figure A1:**
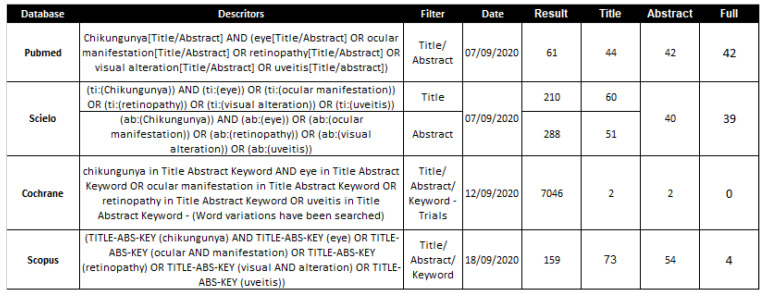
Search strategies used in each database.

A literature search was conducted in the PubMed, Scielo, Cochrane and Scopus databases to review all articles published until September 2020. The following search terms (MeSH) were used: “chikungunya” AND “eye” OR “ocular manifestation” OR “retinopathy” OR “visual alteration” OR “uveitis”. This literature search was conducted in an independent manner by three of the authors (FSP, LSF and VFR). The search strategies and dates used in each database are described in the Appendix A (Figure A1). This systematic review was registered in the International Prospective Register of Systematic Reviews (Prospero/no CRD42020171928) and was developed according with the PRISMA Statement.

### 4.2. Study Selection

All the articles retrieved were screened by three reviewers (FSP, LSF and VFR) for suitability of titles and abstracts and established eligibility and exclusion criteria. Only studies with patients infected by CHIKV and eye alterations were included in the study. Review articles, descriptive studies, studies which did not investigate the ocular manifestations of CHIKV infection, non-human studies, those with patients with other diseases and infections that were not CHIKV, abstracts and studies without relevant data were excluded from the study. Articles which were not retrieved in the bibliography search were manually included in the review.

The manuscripts were independently screened for inclusion by the reviewers who were blinded to each other’s’ decisions. Disagreements between individual judgments were resolved by the corresponding author (ESF). To reduce discrepancies between reviewers in regards of study selection, the exclusion criteria was prioritized as follows: (I) not CHIKV infection, (II) not human study, (III) not ocular manifestation.

### 4.3. Data Extraction

After the selection of the articles, the following data were extracted: the first author’s name, publication year, country in which the study was performed, total number of patients, total numbers of men and women, mean of age (from reported or calculated mean), presence of ocular symptoms (including pain, inflammation, and visual defects) and presence of ocular manifestations (including corneal involvement, conjunctivitis, episcleritis, optic neuritis and uveitis). Furthermore, for each ocular symptom and manifestation, the mean age and numbers of men and women were registered when data were available. Infected patients with no ocular manifestations were used as controls.

### 4.4. Risk of Bias

To evaluate the risk of bias (ROB) of the selected publications, the original Newcastle-Ottawa scale [66] was modified to fit the needs of this review; these modifications were made based in two other modified versions of the Newcastle-Ottawa scale [67,68]. ROB was evaluated by two reviewers (LCMS and FSP).

The modified version used herein, is described in Appendix B. Due to the different characteristics in the design of the studies, no factor was selected as the most important. In total, eight parameters were used in the analysis. A star system was used to determine the risk of bias in each one of the analysed domains. A study received one star in each category if it met the established domain criteria. The maximum number of stars for each study was eight, with studies with less than five stars considered as of high risk, and less than seven stars considered as of moderate risk of bias. The following sources of bias were evaluated: (I) selection bias (sample size, representativeness, responsiveness, and exposure), (II) comparability bias and (III) outcome bias (assessment of outcome, statistical analysis and follow up time).

## 5. Conclusions

This systematic review allowed us to identify the most frequent ocular symptoms and manifestations of CHIKV infection. Ocular pain, inflammation and reduced visual acuity were the most common symptoms, whilst conjunctivitis and optic neuritis were the most common manifestations of the disease. The review findings corroborate previous data that women are the most affected by ocular symptoms and manifestations of CHIKV infection. The few available reports and the moderate-high ROB observed for these studies, highlight the need for further research in the field to gather more substantial and detailed evidence on the link between CHIKV infection, the eye and age/gender. It is important to highlight that it was not possible to determine how long each ocular alteration may last for following infection, as most of the studies brought data from the acute and post-acute phases of the disease. Nonetheless, the data discussed herein emphasize that ocular alterations are meaningful occurrences of CHIKV infection, which can substantially affect the patient’s quality of life. This should bring awareness when dealing with infected patients.

## Figures and Tables

**Table 1:** Prevalence of ocular manifestations in patients diagnosed with CHIKV.

Reference	Origin	Type of Study	Patients	Method of CHIKV Diagnosis	Patients with CHIKV (*n*)	Patients Presenting Ocular Symptoms/Manifestations (*n*)	% of Patients Presenting Ocular Symptoms/Manifestations
KANNAN et al. [13]	India	Cross sectional	All age groups, men and women	Laboratory test/clinical symptoms	354	41	11.6%
ROSE et al. [14]	India	Cross sectional	Adults, men and women	Laboratory test/clinical symptoms	10	10	100%
PERRET et al. [15]	Chile	Cross sectional	Adults, men and women	Laboratory test/clinical symptoms	16	8	50%
ARROYO-ÁVILA et al. [16]	Puerto Rico	Cross sectional	Teens and adults, men and women	Laboratory test/clinical symptoms	172	75	43.6%
VIJAYAKUMAR et al. [17]	India	Cross sectional	All age groups, men and women	Clinical symptoms	1913	419	21.9%
BABU et al. [18]	India	Cross sectional	All age groups, men and women	Laboratory test	2	2	100%
KAWALI et al. [19]	India	Cross sectional	Adults, men and women	Laboratory test/clinical symptoms	6	2	33.3%
PANATO et al. [20]	Brazil	Cross sectional	Adults, men and women	Laboratory test/clinical symptoms	130	32	24.6%
DEEBA et al. [21]	Bangladesh	Cross sectional	All age groups, men and women	Clinical symptoms	1326	817	61.7%
LALITHA et al. [22]	India	Cross sectional	Adults, men and women	Laboratory test	37	37	100%
MAHENDRADAS et al. [23]	India	Cross sectional	Adults, men and women	Laboratory test	9	9	100%
ULLOA-PADILLA et al. [24]	Puerto Rico	Cross sectional	Adults, men and women	Laboratory test	139	42	30.2%
DEL CARPIO-ORANTES et al. [25]	Mexico	Cross sectional	Adults, men and women	Clinical symptoms	1410	151	10.7%
MITTAL et al. [26]	India	Cross sectional	Adults, men and women	Laboratory test/clinical symptoms	14	4	28.6%
KHAREL (SITAULA) et al. [27]	India	Cross sectional	*	Laboratory test	1	1	100%
REZZA et al. [28]	Italy	Cross sectional	All age groups, men and women	Clinical symptoms	205	31	15.1%
HOCHEDEZ et al. [29]	Africa/France	Cross sectional	Adults, men and women	Laboratory test/clinical symptoms	22	1	4.5%
PAUL et al. [30]	India	Cross sectional	Adults, men and women	Laboratory test/clinical symptoms	122	21	17.2%
STAIKOWSKY et al. [31]	France	Cross sectional	Adults, men and women	Clinical symptoms	221	16	7.2%
MANIMUNDA et al. [32]	India	Cross sectional	All age groups, men and women	Laboratory test	203	49	24.1%
CHOPRA et al. [33]	India	Cross sectional	All age groups, men and women	Laboratory test/clinical symptoms	509	36	7.1%
VISHWANATH et al. [34]	India	Cross sectional	Adults, women	Laboratory test	1	1	100%
BABU et al. [35]	India	Cross sectional	*	Laboratory test	1	1	100%
MAHENDRADAS et al. [36]	Germany	Cross sectional	Teens and adults, men and women	Laboratory test/clinical symptoms	3	3	100%
KAWALI et al. [37]	India	Cross sectional	*	Laboratory test/clinical symptoms	5	5	100%

* Data unavailable in the article.

**Table 2:** Prevalence of eye inflammation in patients diagnosed with Chikungunya virus.

Reference	Origin	Patients with CHIKV (n)	Patients Presenting Eye Inflammation			
			Total of Patients (*n*)	Male (*n*)	Female (*n*)	Age (Mean)
KANNAN et al. [13]	India	354	27	*	*	*
VIJAYAKUMAR et al. [17]	India	1913	419	*	*	*
DEEBA et al. [21]	Bangladesh	1326	749	*	*	*
LALITHA et al. [22]	India	37	37	16	21	44.2
MAHENDRADAS et al. [36]	India	9	2	2	0	61.5
ULLOA-PADILLA et al. [24]	Puerto Rico	139	55	*	*	*
MITTAL et al. [26]	India	14	1	*	*	*
KHAREL (SITAULA) et al. [27]	India	1	1	*	*	*
MANIMUNDA et al. [32]	India	203	38	*	*	*
VISHWANATH et al. [34]	India	1	1	1	0	27
BABU et al. [35]	India	1	1	*	*	*
	Total of patients:	3998	Patients with the symptom:	1331	% of patients with the symptom:	33%

* Data unavailable in the original article.

**Table 3:** Prevalence of visual defects in patients diagnosed with Chikungunya virus.

Reference	Origin	Patients with CHIKV (*n*)	Patients Presenting Visual Defects			
			Total of Patients (*n*)	Men (*n*)	Women (*n*)	Age (Mean)
ROSE et al. [14]	India	10	10	3	7	35.8
BABU et al. [35]	India	2	2	0	2	45
KAWALI et al. [37]	India	6	2	*	*	*
DEEBA et al. [21]	Bangladesh	1326	230	*	*	*
LALITHA et al. [22]	India	37	37	*	*	*
MAHENDRADAS et al. [36]	India	9	7	3	4	50.7
ULLOA-PADILLA et al. [24]	Puerto Rico	139	2	*	*	*
MITTAL et al. [26]	India	14	14	5	9	45.8
VISHWANATH et al. [34]	India	1	1	1	0	27
BABU et al. [35]	India	1	1	*	*	*
	Total of patients:	575	Patients with the symptom:	137	% of patients with the symptom:	23%

* Data unavailable in the original article.

**Table 4:** Prevalence of eye pain in patients diagnosed with Chikungunya virus.

Reference	Origin	Patients with CHIKV (*n*)	Patients Presenting Eye Pain			
			Total of Patients (*n*)	Men (*n*)	Women (*n*)	Age (Mean)
KANNAN et al. [13]	India	354	41	*	*	*
ROSE et al. [14]	India	10	10	3	7	35.8
PERRET et al. [15]	Chile	16	8	*	*	*
ARROYO-ÁVILA et al. [16]	Puerto Rico	172	75	*	*	*
MAHENDRADAS et al. [36]	India	9	2	1	1	59
MITTAL et al. [26]	India	14	1	1	0	25
	Total of patients:	1545	Patients with the symptom:	306	% of patients with the symptom:	19%

* Data unavailable in the original article.

**Table 5:** Prevalence of ocular manifestations in patients diagnosed with CHIKV.

Reference	Patients with CHIKV (*n*)	Corneal Involvement				Conjunctivitis				Episcleritis				Optic Neuritis				Uveitis			
		Total of Patients (*n*) Papatiens (*n*)	Women (*n*)	Men (*n*)	Age (Mean)	Total of Patiens (*n*)	Women (*n*)	Men (*n*)	Age (Mean)	Total of Patiens (*n*)	Women (*n*)	Men (*n*)	Age (Mean)	Total of Patiens (*n*)	Women (*n*)	Men (*n*)	Age (Mean)	Total of Patiens (*n*)	Women (*n*)	Men (*n*)	Age (Mean)
ROSE et al. [14]	10													10	3	7	35.8				
BABU et al. [35]	2																	2	0	2	45
KAWALI et al. [37]	6																	2	*	*	*
LALITHA et al. [22]	37	3	*	*	*									8	*	*	*	20	*	*	*
MAHENDRADAS et al. [36]	9									1	1	0	63					8	3	5	51.2
ULLOA-PADILLA et al. [24]	139					27	*	*	*									13			
DEL CARPIO-ORANTES et al. [25]	1410					151	*	*	*												
MITTAL et al. [26]	14													12	5	7	46.5				
KHAREL (SITAULA) et al. [27]	1																	1	*	*	*
REZZA et al. [28]	205					7	*	*	*												
STAIKOWSKY et al. [31]	221					16	*	*	*												
MANIMUNDA et al. [32]	203													49	*	*	*				
VISHWANATH et al. [34]	1																	1	1	0	27
BABU et al. [35]	1																	1	*	*	*
MAHENDRADAS et al. [36]	3																	3	*	*	*
KAWALI et al. [37]	5																	5	*	*	*

* Data unavailable in the original article.

## Data Availability

The datasets used in this study will be made available upon request. Requests should be sent to LCMS (lizianecms@gmail.com).

## Appendix B

**Table A1:** Modified version of the Newcastle-Ottawa scale used in this review.

Sample Representativeness	Sample Size	Non-Respondents	Ascertainment of Exposure	Comparability	Assessment of Outcome	Statistical Analysis	Follow-Up Time
(a) Truly representative of the average population * (b) Somewhat representative of the average the average population* (c) No description	(a) Justified and satisfactory * (b) Not justified	(a) Comparability between respondents and non-responders’ characteristics is established, and the response rate is satisfactory. * (b) The response rate is unsatisfactory, or the comparability between respondents and non-respondents is unsatisfactory. (c) No description of the response rate or the characteristics of the responders and the non-responders	(a) Validated measurement tool * (b) Non-validated measurement tool, but the tool is available or described. * (c) No description of the measurement tool	(a) The study controls for the most important factor (select one). * (b) The study control for any additional factor	(a) Independent blind assessment * (b) Record linkage * (c) Self report (d) No description	(a) The statistical test used to analyse the data is clearly described * (b) The statistical test is described or incomplete	(a) The follow up time was long for the outcome to occur * (b) The follow up time was not long for the outcome to occur

* indicates when a criteria of the domain has been fulfilled.
